# Comparison of the Effects of Microbial Inoculants on Fermentation Quality and Microbiota in Napier Grass (*Pennisetum purpureum*) and Corn (*Zea mays* L.) Silage

**DOI:** 10.3389/fmicb.2021.784535

**Published:** 2022-01-20

**Authors:** Narongrit Jaipolsaen, Siwat Sangsritavong, Tanaporn Uengwetwanit, Pacharaporn Angthong, Vethachai Plengvidhya, Wanilada Rungrassamee, Saowaluck Yammuenart

**Affiliations:** ^1^Physiology and Nutrition Research Team, National Center for Genetic Engineering and Biotechnology, Pathum Thani, Thailand; ^2^Microarray Research Team, National Center for Genetic Engineering and Biotechnology, Pathum Thani, Thailand; ^3^Food Biotechnology Research Team, National Center for Genetic Engineering and Biotechnology, Pathum Thani, Thailand; ^4^Department of Animal and Aquatic Science, Chiang Mai University, Chiang Mai, Thailand

**Keywords:** silage quality, whole-plant corn, Napier grass, lactic acid-producing bacteria, microbiota, bacterial succession

## Abstract

Forage preservation for livestock feeding is usually done by drying the plant material and storing it as hay or ensiling it into silage. During the ensiling process, the pH in the system is lowered by the activities of lactic acid-producing bacteria (LAB), inhibiting the growth of spoilage microorganisms and maintaining the quality of the ensiled product. To improve this process, inoculation of LAB could be used as starter cultures to shorten the ensiling time and control the fermentation process. Here, we compared fermentation quality and bacterial dynamics in two plant materials, whole-plant corn (*Zea mays* L.) and Napier grass (*Pennisetum purpureum*), with and without starter inoculation. The efficacy of *Lactobacillus plantarum*, *L. brevis*, and *Pediococcus pentosaceus* as starter cultures were also compared in the ensiling system. In whole-plant corn, pH decreased significantly, while lactic acid content increased significantly on Day 3 in both the non-inoculated and LAB-inoculated groups. Prior to ensiling, the predominant LAB bacteria were *Weissella*, *Enterococcus*, and *Lactococcus*, which shifted to *Lactobacillus* during ensiling of whole-plant corn in both the non-inoculated and LAB inoculated groups. Interestingly, the epiphytic LAB associated with Napier grass were much lower than those of whole-plant corn before ensiling. Consequently, the fermentation quality of Napier grass was improved by the addition of LAB inoculants, especially *L. plantarum* and a combination of all three selected LAB strains showed better fermentation quality than the non-inoculated control. Therefore, the different abundance and diversity of epiphytic LAB in plant raw materials could be one of the most important factors determining whether LAB starter cultures would be necessary for silage fermentation.

## Introduction

Forages for cattle feed can be preserved by drying the plant materials and storing them as hay or ensiling them into silage or haylage (Silva et al., 2019). One of the main factors affecting preservation quality is the variation in moisture content of different materials and seasons. Tropical forages usually contain high moisture content and always grow out during the rainy season; therefore, making silage can be a more practical method of forage preservation than making hay. Ensiling is a method of forage preservation based on bacterial fermentation, such as by lactic acid-producing bacteria (LAB) (Oude Elferink et al., 2000; Fabiszewska et al., 2019). During the ensiling process, the pH in the system decreases due to the activities and abundance of LAB, which come from epiphytic bacteria under anaerobic conditions. When the pH decreases to a level where the growth of undesirable microorganisms such as *Clostridium* spp., yeast and molds is inhibited, good quality silage is obtained (Cai et al., 1998; Musen et al., 2014). In addition, previous studies have reported that inoculation of LAB can help shorten the fermentation process and ensiling time and directly maintain silage quality by increasing lactic acid content and reducing butyric acid (Chen et al., 2017). Nevertheless, conflicting results regarding the need for starter cultures have been observed in ensiling different forage crops. For example, a meta-analysis on the effects of LAB- inoculation on silage fermentation shows that LAB-inoculation could improve the fermentation quality of grasses and legumes, but not in corns, sorghum, and sugarcane (Oliveira et al., 2017).

The silage microbiota contains beneficial microorganisms such as LAB and spoilage microorganisms such as mold and yeast (Kim et al., 2021). During the fermentation process, the pH in the system decreases due to the activities of the abundance of LAB originated from epiphytic bacteria under anaerobic conditions. When the pH decreases to a point where undesirable microbes such as *Clostridium* spp., yeast, and mold are not able to survive, consequently a good silage quality is achieved (Musen et al., 2014). Therefore, the abundance and species of epiphytic bacteria in plant materials are crucial for the spontaneous fermentation process and microbial succession in silage production (Fabiszewska et al., 2019). The members of the epiphytic microbiota and their variations are a critical factor in determining whether LAB inoculation was necessary for silage production (Lin et al., 1992). In the past, beneficial microbial communities were identified simply based on a culture-dependent approach, such as isolating strains on the selective media (e.g., MRS media) for LAB content analysis. However, this method has not been able to characterize and identify all microbial communities in silage because some microbial species do not form colonies or cannot be cultured (Xu et al., 2017). Recent advances in sequencing technology have made it possible to perform microbial population analyses to obtain a comprehensive view of community structures, including the unculturable microorganisms (Cao et al., 2017; Bokulich et al., 2020). Direct analysis of DNA purified from samples has proven useful for taxonomic identification of the overall microbial ecologies in the samples (Tennant et al., 2017; Guo et al., 2018).

Among the various forages, the silage of whole-plant corn (*Zea mays* L.) has been used worldwide and has become an important feed due to its high digestible nutrients and protein efficiency (Ferraretto et al., 2018). Alternatively, Napier grass (*Pennisetum purpureum*) is widely used in tropical regions due to its availability and high biomass (Muia, 2000; Rusdy, 2016). Napier grass is fast-growing, and its yield can range from 3.41 to 4.74 tons DM/ha/cut with a cutting age of 42 days (Haryani et al., 2018). Under irrigation system in Ethiopia, it has been reported that Napier grasses could yield 17.74–16.89 tons DM/ha/cut at a cutting age of 4.5 months (Getiso and Mijena, 2021). The most important factors contributing to silage consistency and quality are the abundance and composition of epiphytic bacteria and the content of fermentable sugars which can vary depending on the plant species (Fabiszewska et al., 2019). The lower abundance of epiphytic LAB in combination with low fermentable carbohydrates can be overcome by adding starter cultures to inhibit the growth of the undesirable microorganisms (Kim et al., 2021).

Here, we investigated the importance of LAB inoculants on the fermentation quality of silages prepared from two commonly used forages: (i) whole-plant corn (*Z. mays* L.) and (ii) Napier grass (*P. purpureum*). The effects of starter inocula on fermentation quality and microbial diversity during the fermentation were determined to compare their effectiveness for silage preservation in the two different crops to obtain a high-quality product.

## Materials and Methods

### Bacterial Strains and Growth Conditions

*Lactobacillus plantarum* J39 was isolated from the ensiled total mixed ration containing baby corn husk as the main roughage in our previous study (Jaipolsaen et al., 2019). *L. brevis* BCC42336 and *Pediococcus pentosaceus* TBRC7603 were obtained from the Thailand Bioresource Research Center, Thailand, and were isolated from fermented Shona cabbage (*Cleome gynandra*) and fermented bamboo shoot (*Dendrocalamus asper*), respectively. To prepare starter inocula, all strains were grown in De Man Rogosa and Sharpe (MRS) broth (Lactobacilli MRS Broth, Difco & BBL, United States) at 37°C for 18 h.

### Preparation of Silage

An aerial part of the whole-plant corn (*Z. mays* L.) and Napier grass (*P. purpureum*) were used as raw plant materials. Both were harvested from local farms in Saraburi, Thailand (14° 38′ 02.5′ N, 101° 11′ 00.3″E). The fresh Napier grass and an above-ground portion of the corn plant were chopped to a length of 2–3 cm and anaerobically packed into 10 × 15-inch polyethylene bags for silage production. Using a Completely Randomized Design (CRD), silage treatments were divided into five groups that included plant material: (*i*) no inoculation with starter cultures (Control), (*ii*) inoculated with *L. plantarum* J39 at 10^7^ CFU/g fresh weight (T1), (*iii*) inoculated with *L. brevis* BCC42336 at 10^7^ CFU/g fresh weight (T2), (*iv*) inoculated with *P. pentosaceus* TBRC7603 at 10^7^ CFU/g fresh weight (T3), and (v) inoculated with a combination of *L. plantarum* J39, *L. brevis* BCC42336 and *P. pentosaceus* TBRC7603 at 10^7^ CFU/g fresh weight in a 2:1:1 ratio (T4) (Supplementary Figure 1). Each LAB-inoculated group was sprayed with a designated starter culture and mixed thoroughly to ensure homogeneity, and the non-inoculated control group was sprayed with sterile normal saline. Each experimental diet was thoroughly mixed before packing each of 2 kg of freshly chopped Napier grass or freshly chopped whole-plant corn of each individual treatment group into the polyethylene bags. After packing, all bags were vacuum-sealed using a small automatic vacuum sealing machine (Goodluck sealing machine©, model VS100S, China). All bags were stored at room temperature. The samples were collected in a three-point of mixing tray on Day 0 (D0), Day 3 (D3), and Day 7 (D7) after ensiling (Supplementary Figure 1). Then, the collected samples were pooled and placed in small polyethylene bags, vacuum-sealed, and stored at –20°C for fermentation qualities analyses. At the same time, a small portion of the collected samples was immediately ground to a small size (0.5–2 mm) using a laboratory-scale blender. The ground samples were stored in a –80°C freezer for DNA extraction.

### Fermentation Quality, Monosaccharides, and Starch Contents Analyses

The pH values of the fermented feeds were measured using a pH meter (pH Bench F20-Meter with Inlab Semi-Micro pH probe, Mettler-Toledo, Greifensee, Switzerland). For organic acid analysis, 12.5 g of the fresh samples were chopped and homogenized with 100 mL of 0.01 M H_2_SO_4_ in a lab-blender stomacher (Seward Laboratory, London). Then, one mL of the supernatant of each sample was filtered through a 0.45 μm nylon membrane syringe filter (Anpel laboratory technologies, Shanghai, China). Organic acid profiles were then analyzed using an HPLC–UV system (Alliance^®^ HPLC e2695 Separations Module and 2998 Photodiode Array Detector, Water, Massachusetts, United States), with an Aminex HPX-87H HPLC column (BIO-RAD^®^, California, United States). Analyses were performed isocratically under the conditions of 0.6 mL/min, and 65°C with 0.01 M H_2_SO_4_ as the mobile phase.

For carbohydrate content analysis, fresh samples were dried at 60°C for 48 h. One gram of the dried sample was homogenized with 10 mL of distilled water for 1 h. After homogenization and centrifugation, the supernatant was filtered through a 0.45 μm nylon membrane syringe filter. Monosaccharides were analyzed using an HPLCUV system (Shimadzu; SIL-10A, Tokyo, Japan). Filtered distilled water in an Aminex HPX-87P HPLC column (BIO-RAD^®^, California, United States) was used as the mobile phase. Total starch was analyzed using the K-TSTA 07/11 Megazyme kit according to AOAC International Method 996.11 (AOAC et al., 2007).

Silage quality was evaluated using the following equation Flieg (1938) cited in He et al. (2021).

Flieg’s score = 220 + (2 x%Dry matter–15)–40 × pH

According to the equation, silage was classified as poor, bad, low, medium, and good if the score values were below 20, 21–40, 41–60, 61–80, and above 80, respectively.

### DNA Isolation and Purification

Each silage sample was homogenized to fine powder using a blender. An equal amount of 250 mg of each ground sample was used for DNA extraction using the DNeasy^®^ PowerSoil Kit (Qiagen^®^, Germany) according to the manufacturer’s protocol. DNA samples were purified and concentrated using the Genomic DNA Clean and Concentrator (Zymo Research^®^, United States) according to the manufacturer’s protocol. DNA purity was quantified using NanoDrop 8000 spectrophotometer (Thermo Fisher Science, United States) and stored at –20°C until use.

### 16S rRNA Amplicon Preparation and Sequencing

The V3 and V4 regions were amplified with the following primer pairs; forward primer (5′ TCGTCGG CAGCGTCAGATGTGTATAAGAGACAGCCTACGGGNGGC WGCAG 3′) and reverse primer (5′ GTCTCGTG GGCTCGGAGATGTGTATAAGAGACAGGACTACHVGGGT ATCTAATCC 3′) (Klindworth et al., 2013). The 16S rRNA amplicon library was constructed by using proofreading Q5^®^ High-Fidelity DNA polymerase (New England Biolabs^®^, United States) with the following PCR cycle parameters: 98°C for 3 min, then 25 cycles of 98°C for 30 s, 54°C for 30 s and 72°C for 30 s, and the final extension was performed at 72°C for 2 min. PCR amplicons were purified using QIAquick^®^ Gel Extraction Kit (Qiagen^®^, Germany) according to the manufacturer’s protocol. All 16S rRNA amplicon libraries were subjected to Illumina MiSeq next-generation sequencing (Macrogen Inc., Korea).

### Bioinformatic Analysis of Sequencing Data

Raw sequences were qualitatively filtered and analyzed using TrimGalore (Krueger, 2015) and FastQC (Simon, 2010). Sequences longer than 200 bp containing homopolymers less than 6 bp, and a mean sequence quality score greater than 20 passed quality control criteria. Sequences were subsequently analyzed using Qiime2 (Hall and Beiko, 2018) and DADA2 (Callahan et al., 2016) to remove chimeras and dereplicate DNA reads, and call amplicon sequence variants (ASVs). ASVs were assigned to the lowest possible taxonomic rank using the BLAST + consensus taxonomy classifier against SILVA version 132. Data analysis was performed using Phyloseq (McMurdie and Holmes, 2013) under the R 3.5.2 environment (Rstudio Team, 2015; R Core Team, 2020). Statistical significance of beta diversity between groups was determined using Permutational Multivariate Analysis of Variance (PERMANOVA) (vegan package, function adonis) (Anderson, 2001). Differences in microbiome composition were estimated using the Kruskal-Wallis *H*-test, and all *p*-values were corrected using the false discovery rate method (Benjamini and Hochberg, 1995). A false discovery rate (FDR) of < 0.05 was considered statistically significant.

### Statistical Analysis

The statistical analyses were performed using the R 3.6.1 environment. Water-soluble carbohydrate contents and fermentation qualities were statistically analyzed using Analysis of Variance (ANOVA) according to the CRD design. Significant differences (*P* < 0.05) were calculated by Tukey’s test (Steel and Torrie, 1980). Permutational analysis of variance (PERMANOVA) statistical test was used to compare beta diversity among microbial communities (Oksanen et al., 2019).

## Results

### Fermentation Quality, Monosaccharides, and Starch Contents

Whole-plant corn and Napier grass were fermented with or without starter inoculation. The plant materials were inoculated to yield 10^7^ CFU/g fresh weight of different starter inocula, *L. plantarum* J39 (T1), *L. brevis* BCC42336 (T2), *P. pentosaceus* TBRC7603 (T3), and a combination of *L. plantarum* J39, *L. brevis* BCC42336 and *P. pentosaceus* TBRC7603 (T4). At the same time, normal saline was sprayed to the non-inoculated control group (Supplementary Figure 1). Silages were sampled at D0, D3, and D7 to determine fermentation quality. To determine content of fermentable carbohydrates before ensiling, total sugars and individual monosaccharides, including glucose, xylose, galactose, fructose, and starch content, were determined to compare the initial sugar contents in the different plant raw materials (Table 1). The contents of total sugars, glucose, galactose, and fructose were significantly higher in whole-plant corn than in Napier grass. In contrast, xylose contents were significantly higher in Napier grasses. Whole-plant corn also contained significantly higher levels of starch than Napier grass.

**TABLE 1:** Sugar content analysis in freshly cut the freshly cut aerial part of whole-plant corn and Napier grass.

Item	Material		SEM	*P*-value
	
	Whole-plant corn	Napier grass		
Total sugar (%DM)	11.76	0.32	2.56	>0.01
Glucose (%DM)	6.57	0.12	1.44	>0.01
Xylose (%DM)	0.07	0.09	0.01	>0.01
Galactose (%DM)	0.36	0.04	0.07	>0.01
Fructose (%DM)	4.77	0.07	1.05	>0.01
Starch (%DM)	13.66	2.27	2.57	>0.01

*All analyses were carried out in triplicate. P-value < 0.05 was considered as statistically significant difference. SEM, standard error of the mean.*

Dry matter content (DM), pH, and organic acid contents were also determined (Tables 2, 3). In whole-plant corn silage, the DM content was significantly decreased in all groups after 7 days of fermentation; however, no treatment effects were observed (Table 2). The pH values of corn silage were significantly decreased in all treatment groups and reached the plateau on D3 after ensiling. In addition, pH values did not differ significantly between treatments within the same days of ensiling. Lactic acid levels were not significantly different between treatments on D3 and D7 after fermentation. In contrast, lactic acid concentrations were significantly higher in all treatments on D7 compared to D3 after fermentation. Butyric acid concentration was undetectable in all treatment groups at both D3 and D7 after ensiling. On the other hand, pyruvic acid content of fresh whole-plant corn (D0) was highest in all treatment groups but decreased significantly after the start of ensiling. Our results clearly showed that starter inocula did not provide significant advantages for the fermentation of corn silage.

**TABLE 2:** Fermentation quality of whole-plant corn silage during the ensiling process.

Item	Day	Treatments					SEM	*P*-value
		
		Control (0.85% NaCl)	T1 (*L. plantarum* J39)	T2 (*L. brevis* BCC42336)	T3 (*P. pentosaceus* TBRC7603)	T4 (combination)		
DM (%)	0	22.68^x^	22.59^x^	22.48^x^	22.58^x^	22.43^x^	0.04	0.18
	3	21.61^y^	21.15^y^	21.76^y^	21.78^y^	21.06^y^	0.11	0.10
	7	19.74^z^	19.36^z^	19.90^z^	19.71^z^	19.22^z^	0.08	0.09
SEM		0.39	0.43	0.36	0.40	0.42		
*P*-value		<0.01	<0.01	<0.01	<0.01	<0.01		
pH	0	5.43^x^	5.44^x^	5.44^x^	5.44^x^	5.43^x^	0.00	0.90
	3	3.56^y^	3.57^y^	3.57^y^	3.56^y^	3.55^y^	0.00	0.29
	7	3.55^y^	3.52^y^	3.56^y^	3.55^y^	3.54^y^	0.00	0.32
SEM		0.25	0.25	0.25	0.25	0.25		
*P*-value		<0.01	<0.01	<0.01	<0.01	<0.01		
Lactic acid	0	ND	ND	ND	ND	ND		
(%DM)	3	7.57^y^	7.34^y^	7.72^y^	7.77^y^	7.42^y^	0.18	0.93
	7	9.89^x^	9.52^x^	9.92^x^	9.53^x^	9.71^x^	0.12	0.77
SEM		0.49	0.47	0.46	0.37	0.55		
*P*-value		<0.01	<0.01	<0.01	<0.01	<0.01		
Butyric acid	0	ND	ND	ND	ND	ND	N/A	N/A
(%DM)	3	ND	ND	ND	ND	ND	N/A	N/A
	7	ND	ND	ND	ND	ND	N/A	N/A
SEM		N/A	N/A	N/A	N/A	N/A		
*P*-value		N/A	N/A	N/A	N/A	N/A		
Pyruvic acid	0	21.96^x^	23.08^x^	22.90^x^	23.02^x^	22.16^x^	0.21	0.34
(%DM)	3	4.25^by^	4.22^by^	6.44^ay^	6.14^ay^	4.43^by^	0.23	<0.01
	7	2.90^bz^	2.76^bz^	4.37^az^	4.57^az^	3.14^bz^	0.18	<0.01
SEM		2.46	2.32	2.36	2.35	2.46		
*P*-value		<0.01	<0.01	<0.01	<0.01	<0.01		

*No bacterial inoculum was added to the control group and four different bacterial strains, Lactobacillus plantarum J39 (T1), L. brevis BCC42336 (T2), Pediococcus pentosaceus TBRC7603 (T3), and a combination of L. plantarum J39, L. brevis BCC42336 and P. pentosaceus TBRC7603 (T4) were inoculated prior to the ensiling process. All analyses were carried out in four replicates (two replicates of each sample were collected; A and B). P-value < 0.05 was considered as statistically significant difference.*

*^a,b^Superscript letters demonstrated significant differences at P < 0.05 within the same row.*

*^x,y,z^ Superscript letters demonstrated significant differences at P < 0.05 within the same column. ND, the values are not detected; N/A, the values are not available; SEM, standard error of the mean.*

**TABLE 3:** Fermentation quality of Napier grass silage during the ensiling process.

Item	Day	Treatments					SEM	*P*-value
		
		Control (0.85% NaCl)	T1 (*L. plantarum* J39)	T2 (*L. brevis* BCC42336)	T3 (*P. pentosaceus* TBRC7603)	T4 (combination)		
DM (%)	0	25.99	25.99	25.96	25.96	25.9	0.21	1.00
	3	25.96	25.45	25.57	25.79	25.64	0.27	0.99
	7	25.99	25.58	25.43	25.36	25.65	0.20	0.93
SEM		0.19	0.39	0.29	0.33	0.39		
*P*-value		0.99	0.90	0.82	0.79	0.96		
pH	0	5.77^x^	5.77^x^	5.75^x^	5.76x	5.76x	0.01	0.91
	3	4.78^ay^	3.96^dy^	4.32^by^	4.16^cy^	3.93^dy^	0.10	<0.01
	7	4.13^az^	3.82^dz^	4.07^bz^	3.98^cz^	3.84^dz^	0.04	<0.01
SEM		0.30	0.40	0.33	0.36	0.40		
*P*-value		<0.01	<0.01	<0.01	<0.01	<0.01		
Lactic acid	0	ND	ND	ND	ND	ND		
(%DM)	3	2.76^cy^	6.36^ay^	4.10^by^	4.68^by^	6.01^ay^	0.31	<0.01
	7	6.58^cx^	8.30^ax^	6.89^bcx^	6.98^bx^	8.11^ax^	0.16	<0.01
SEM		0.72	0.37	0.53	0.44	0.44		
*P*-value		<0.01	<0.01	<0.01	<0.01	<0.01		
Butyric acid	0	ND	ND	ND	ND	ND	N/A	N/A
(%DM)	3	ND	ND	ND	ND	ND	N/A	N/A
	7	ND	ND	ND	ND	ND	N/A	N/A
SEM		N/A	N/A	N/A	N/A	N/A		
*P*-value		N/A	N/A	N/A	N/A	N/A		
Pyruvic acid	0	ND	ND	ND	ND	ND		
(%DM)	3	0.16^a^	0.10^by^	0.10^bx^	0.14^ay^	0.09^b^	0.01	<0.01
	7	0.19^a^	0.13^bx^	0.05^dy^	0.17^ax^	0.10^c^	0.01	<0.01
SEM		0.01	0.01	0.01	0.01	0.00		
*P*-value		0.12	0.01	<0.01	<0.01	0.12		

*No bacterial inoculum was added to the control group and four different bacterial strains, Lactobacillus plantarum J39 (T1), L. brevis BCC42336 (T2), Pediococcus pentosaceus TBRC7603 (T3), and a combination of L. plantarum J39, L. brevis BCC42336 and P. pentosaceus TBRC7603 (T4) were inoculated prior to the ensiling process. All analyses were carried out in four replicates (two replicates of each sample were collected; A and B). P-value < 0.05 was considered as statistically significant difference.*

*^a,b,c,d^Superscript letters demonstrated significant differences at P < 0.05 within the same row. ^x,y,z^Superscript letters demonstrated significant differences at P < 0.05 within the same column. ND, the values are not detected; N/A, the values are not available; SEM, standard error of the mean.*

In contrast to whole-plant corn ensiling, starter inocula were beneficial for ensiling Napier grass (Table 3). All starter inocula were able to significantly improve the fermentation quality of Napier grass silage (Table 4). The pH values of Napier grass silage in the inoculated treatments (T1–T4) were significantly lower than those of the control group on D3 and D7 after ensiling. In addition, the pH values of Napier grass silage T1 and T4 groups had the lowest pH values on both D3 and D7 after ensiling. Lactic acid concentration in inoculated Napier grass was significantly higher than in the control group. In particular, Napier grass silage T1 and T4 groups had the highest lactic acid levels on D3 and D7 after ensiling. Butyric acid was not detected in all treatments at all-time points. Unlike whole-plant corn, pyruvic acid was not detected in fresh Napier grass (D0); however, a low concentration of pyruvic acid was detected at D3 and D7 after fermentation (Table 3). Interestingly, all starter inocula in this study showed a positive effect on Napier grass ensiling but not on whole-plant corn ensiling (Table 4).

**TABLE 4:** Silage quality classification of whole-plant corn and Napier grasses silage after 3 and 7 days of the ensiling process from Flieg’s evaluation system.

Materials	Day	Treatments					SEM	*P*-value
		
		Control (0.85% NaCl)	T1 (*L. plantarum* J39)	T2 (*L. brevis* BCC42336)	T3 (*P. pentosaceus* TBRC7603)	T4 (combination)		
Whole-plant corn	3	105.71	104.51	105.71	106.06	105.13	0.28	0.45
	7	102.48	102.83	102.50	102.12	101.75	0.14	0.11
Napier grass	3	65.92^d^	97.60^a^	83.24^c^	90.25^b^	98.98^a^	2.78	<0.01
	7	91.87^c^	103.25^a^	93.27^c^	96.61^b^	102.89^a^	1.11	<0.01

*No bacterial inoculum was added to the control group and four different bacterial strains, Lactobacillus plantarum J39 (T1), L. brevis BCC42336 (T2), Pediococcus pentosaceus TBRC7603 (T3), and a combination of L. plantarum J39, L. brevis BCC42336 and P. pentosaceus TBRC7603 (T4) were inoculated prior to the ensiling process. All analyses were carried out in four replicates (two replicates of each sample were collected; A and B). P-value < 0.05 was considered as statistically significant difference.*

*^a,b,c,d^Superscript letters demonstrated significant differences at P < 0.05 within the same row. SEM = standard error of the mean. The quality classification was considered from numerical Flieg’s score, which to be a very bad, poor, low, medium and Good quality when it had score value with under 20, 21–40, 41–60, 61–80, and over than 80 respectively.*

### Bacterial Community Dynamics During Ensiling of Whole-Plant Corns

**FIGURE 1:**
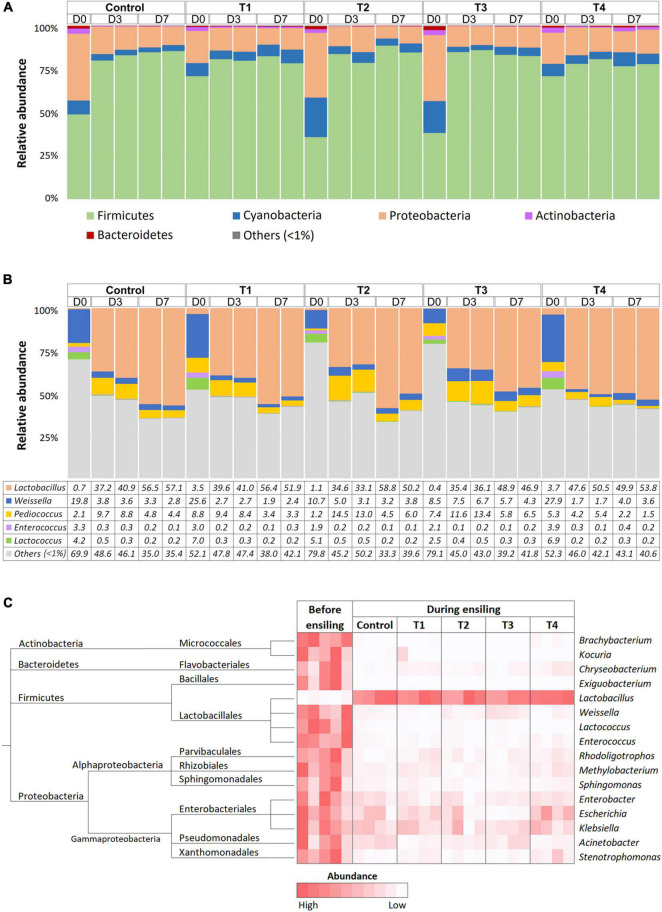
Bacterial profiles of whole-plant corn silage in five treatment groups, which were a non-inoculated control group (control), and the groups inoculated with following starter inocula, *Lactobacillus plantarum* J39 (T1), *L. brevis* BCC42336 (T2), *Pediococcus pentosaceus* TBRC7603 (T3), and a combination of *L. plantarum* J39, *L. brevis* BCC42336, and *P. pentosaceus* TBRC7603 (T4). Silage was sampling on Day 0 (D0), Day 3 (D3), and Day 7 (D7) of ensiling. **(A)** Relative abundance of bacterial phyla. **(B)** Relative abundance of lactic acid producing bacteria genera. **(C)** Differences in bacterial profiles identified by Kruskal-Wallis *H*-test at ASV level. Taxonomic tree and heatmaps of microbiota based on the results of the comparison between treatment and control at D3 and D7 in corn plant silages. The significant ASVs with an adjusted *p*-value < 0.05 were shown in the heatmap.

Bacterial composition was monitored at D0, D3, and D7 time points of ensiling using 16S amplicon sequencing. On average, 97,943 ± 16,360 and 103,285 ± 13,114 reads were obtained from whole-plant corn and Napier grass silage, respectively (Supplementary Table 1). The rarefaction curves of all sampling time points reached a plateau, indicating sufficient sequencing depth to describe bacterial profiles during plant ensiling (Supplementary Figure 2). Bacterial taxonomy was classified based on amplicon sequence variants (ASVs) (Supplementary Table 2). Bacterial community structures were determined in five treatment groups: control, T1, T2, T3, and T4 at D0, D3, and D7 of ensiling (Figure 1). Bacterial community profiles in whole-plant corn silages were dominated by phyla Firmicutes, Proteobacteria, and Cyanobacteria (Figure 1A). During the ensiling process of whole-plant corns, the relative abundance of Firmicutes showed increasing trends. In contrast, the relative abundance of Proteobacteria and Cyanobacteria decreased in all groups compared to D0. Silage quality could be indicated by the amount of lactic acid produced by LAB. Here, the relative abundance of LAB increased in both the non-inoculated control and the inoculated groups (T1, T2, T3, and T4), and the dominant genus observed on D3 and D7 of whole-plant corn silage was *Lactobacillus* (Figure 1B). *Weissella*, *Pediococcus*, *Enterococcus*, and *Lactococcus* were other predominant lactic acid bacteria that showed decreasing abundance, while dominance shifted to Lactobacillus on D3 and D7. Interestingly, the relative abundance of *Pediococcus* was higher on D3 than on D0 before decreasing on D7 in all groups. Notably, the whole-plant corn silage inoculated with *Pediococcus* (T3) was dominated by the genus *Lactobacillus*, as observed on D3 and D7 of fermentation. Nevertheless, the LAB patterns were mostly similar in the non-inoculated control and inoculated groups. In addition, Kruskal-Wallis *H* was used to determine significant differences in bacterial abundance before (D0) and during ensiling (D3 and D7) (Figure 1C). The results showed that the abundance of *Lactobacillus* increased significantly in all groups, while the abundance of Actinobacteria (*Brachybacterium* and *Kocuria*), Bacteroidetes (*Chryseobacterium*), Firmicutes (*Exiguabacterium*, *Weisella*, *Lactococcus*, and *Enterococcus*), Proteobacteria (*Rhodoligotrophas*, *Methylobacterium*, *Sphingomonas*, *Enterobacter*, *Escherichia*, *Klebsiella*, *Acinetobacter*, and *Stenotrophomonas*) were significantly reduced during ensiling.

### Bacterial Community Dynamics During Ensiling of Napier Grasses

**FIGURE 2:**
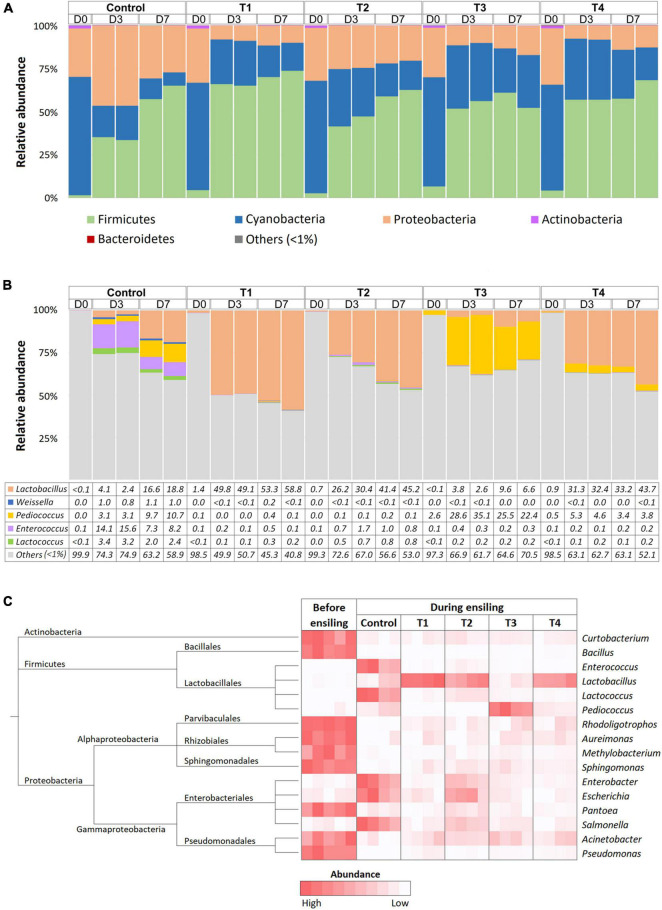
Bacterial profiles of Napier grass silage in five treatment groups, which were a non-inoculated control group (control), and the groups inoculated with following starter inocula, *Lactobacillus plantarum* J39 (T1), *L. brevis* BCC42336 (T2), *Pediococcus pentosaceus* TBRC7603 (T3), and a combination of *L. plantarum* J39, *L. brevis* BCC42336, and *P. pentosaceus* TBRC7603 (T4). Silage was sampling on Day 0 (D0), Day 3 (D3), and Day 7 (D7) of ensiling. **(A)** Relative abundance of bacterial phyla. **(B)** Relative abundance of lactic acid producing bacteria genera. **(C)** Differences in bacterial profiles identified by Kruskal-Wallis *H*-test at ASV level. Taxonomic tree and heatmaps of microbiota based on the results of the comparison between treatment and control at D3 and D7 in Napier grass silages. The heatmaps show representative ASVs with an adjusted *p*-value < 0.05.

Bacterial communities in Napier grasses were dominated by three main phyla, Cyanobacteria, Firmicutes, and Proteobacteria, while Actinobacteria dominated only at D0 (Figure 2A). Cyanobacteria were the dominant population in the pre-silage (D0) with an abundance of over 70%. After ensiling, the abundance of Cyanobacteria decreased in all groups. Firmicutes became the dominant group during ensiling in both the non-inoculated control group and the LAB-inoculated groups (T1, T2, T3, and T4). Interestingly, the abundance of *Proteobacteria* was reduced in the four LAB-inoculated groups but not in the non-inoculated control group. The relative abundance of the major LAB genera, *Lactobacillus*, *Weissella*, *Pediococcus*, *Enterococcus*, and *Lactococcus*, were determined and compared (Figure 2B). The dynamic changes of LAB among Napier grass silages were strikingly observed in the LAB-inoculated groups, while little change in the abundance of LAB was observed in the non-inoculated control group. From D0 to D3, the relative abundance of *Lactobacillus* in T1, T2, and T4 shifted sharply from 1.4 to ∼50%, from 0.7 to ∼30%, and from 0.9 to 31%, respectively. As expected, T3, the Napier grass inoculated with *P. pentosaceus*, had the highest abundance of *Pediococcus* at D3 and D7 compared to the other LAB-inoculated groups. Our observations suggest that starter culture strains influenced LAB diversity in ensiling Napier grass. Accordingly, Kruskal-Wallis *H*-test showed a significant increase of *Lactobacillus* in T1, T2, and T4, while *Pediococcus* was significantly present in T3 (Figure 2C). In contrast to the LAB-inoculated groups, the non-inoculated group was not dominated by a single group of LAB but showed a greater diversity of LAB, namely *Enterococcus*, *Lactococcus*, and *Pediococcus*. Both control and LAB-inoculated groups (T1, T2, T3, and T4) showed significant reduction of Proteobacteria (*Rhodoligotrophos*, *Aureimonas*, *Methylobacterium*, *Sphingomonas*, *Pantoea*, *Acinetobacter*, and *Pseudomonas*) and Actinobacteria (*Curtobacterium*). However, the abundance of *Enterobacter*, *Escherichia*, and *Salmonella*, which are potentially pathogenic bacterial groups was significantly increased in the control group during Napier grass ensiling.

### Effects of Starter Inoculation on Bacterial Diversity in Silages

**FIGURE 3:**
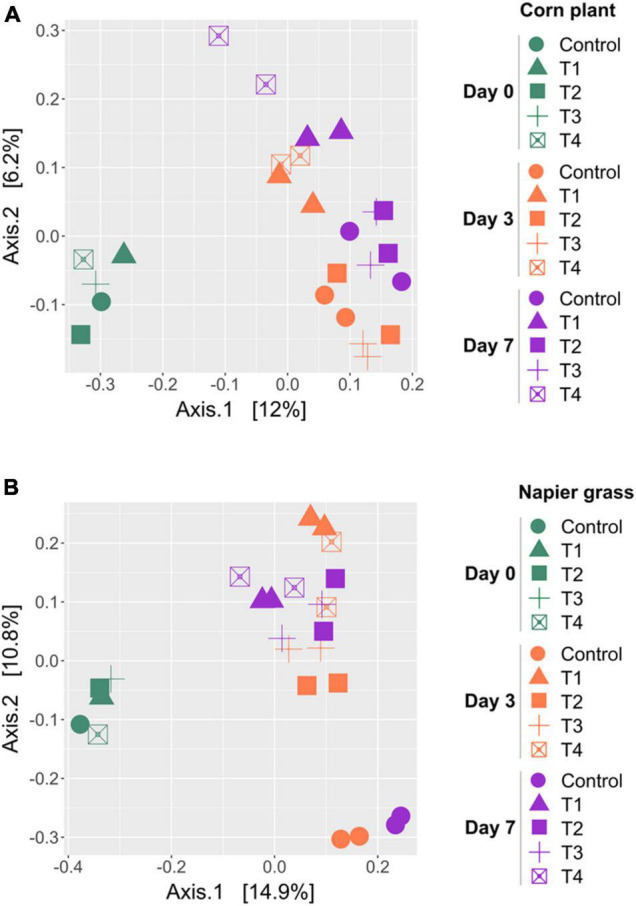
Principal coordinates analysis (PCoA) of bacterial community profiles in silages made from whole-plant corns **(A)** and Napier grasses **(B)**. The analysis was carried out based on unweighted-UniFrac distance matrix, and individual samples were color-coordinated according to sampling day of the ensiling, in which green represents Day 0 (D0), orange represents Day 3 (D3), and purple represents Day 7 (D7). A control group (circle), and the four groups with potential lactic acid bacteria starter cultures, *Lactobacillus plantarum* J39 (T1, triangle), *L. brevis* BCC42336 (T2, square), *Pediococcus pentosaceus* TBRC7603 (T3, plus sign), and a combination of *L. plantarum* J39, *L. brevis* BCC42336, and *P. pentosaceus* TBRC7603 (T4, opened square) were compared.

A principal coordinate analysis (PCoA) based on the unweighted-UniFrac was used to compare the similarity of bacterial profiles during ensiling (Figure 3). PCoA showed a clear separation of the bacterial composition of the whole-plant corn silages at the beginning (D0), D3 and D7 of ensiling (Figure 3A). The bacterial profiles of the control and inoculated groups (T1, T2, T3, and T4) were similar at D0 and showed a shift at D3 and D7. Bacterial profiles of whole-plant corn were similar at D3 and D7 despite the addition of inoculants. In contrast, the PCoA of Napier grass silages showed three distinct clusters (Figure 3B). The bacterial profiles of all five groups were similar at D0. During ensiling, the bacterial communities of the control and inoculated groups (T1, T2, T3, and T4) were clearly separated at D3 and D7, indicating that starter inoculation affected the bacterial diversity of Napier grass silages. Consistently, Permutational Multivariate Analysis of Variance (PERMANOVA) of bacterial communities based on the unweighted-UniFrac distance matrix was used to determine the factors affecting bacterial profiles in the different sample groups (Table 5). Bacterial profiles in whole-plant corn and Napier grass were significantly different (*P* < 0.05). All four formulations of LAB starter cultures had a significant effect on bacterial diversity in Napier grass silage (*P* < 0.05), while only a combination of LAB consortia containing *L. plantarum*, *L. brevis*, and *P. pentosaceus* (T4), showed a significant effect on bacterial profiles in whole-plant corn silage. In Napier grass, the different LAB strains had a significant effect on bacterial communities (*P* < 0.05), except that the group inoculated with a combination of LAB consortia (T4) had similar bacterial profiles to the group inoculated with a single strain, namely *L. plantarum* (T1) and *P. pentosaceus* (T3).

**TABLE 5:** Permutational Multivariate Analysis of Variance (PERMANOVA) on bacterial community associated with corn plant and Napier grass silages.

Factor		*F* model	*R* ^2^	*P*-value
**Plant material source**				
Whole-plant corn vs. Napier grass		4.40	0.36	**0.006**
**Non-inoculated vs. Inoculated ensiling**				
Whole-plant corn	Control vs. T1	1.02	0.15	0.352
	Control vs. T2	0.98	0.14	0.606
	Control vs. T3	0.91	0.13	0.905
	Control vs. T4	1.29	0.18	**0.042**
Napier grass	Control vs. T1	2.59	0.30	**0.034**
	Control vs. T2	2.16	0.26	**0.031**
	Control vs. T3	2.09	0.26	**0.026**
	Control vs. T4	2.57	0.30	**0.031**
**Types of starter inoculants**				
Whole-plant corn	T1 vs. T2	1.22	0.17	**0.024**
	T1 vs. T3	1.24	0.17	**0.029**
	T1 vs. T4	1.04	0.15	0.174
	T2 vs. T3	1.04	0.15	0.243
	T2 vs. T4	1.43	0.19	**0.022**
	T3 vs. T4	1.44	0.19	**0.035**
Napier grass	T1 vs. T2	1.71	0.22	**0.025**
	T1 vs. T3	1.66	0.22	**0.034**
	T1 vs. T4	1.30	0.18	0.089
	T2 vs. T3	1.54	1.54	**0.037**
	T2 vs. T4	1.57	0.21	**0.030**
	T3 vs. T4	1.26	0.17	0.063

*Analysis was carried out based on unweighted-UniFrac distance matrix, and the samples from before ensiling (Day 0), and during ensiling (Day 3 and Day 7) were compared.*

*Values in bold are statistically significant (P-value < 0.05).*

## Discussion

Understanding bacterial dynamics during ensiling is important for improving the fermentation process, quality, and stability of forage silage. The objective of this study was to analyze the fermentation quality and bacterial dynamics of whole-plant corn and Napier grass silages with and without inoculants to determine the requirement of starter cultures in the ensiling process. The inoculated Napier silage achieved the desired fermentation quality faster than the non-inoculated silage; in particular, the inoculation of *L. plantarum* J39 showed a significantly lower pH and higher lactic acid content compared to the control groups. In contrast, the whole-plant corn was more readily fermented into high fermentation quality silage with and without the addition of lactic acid bacteria (LAB) starter cultures.

The LAB are able to enhance homo-lactic fermentation, producing a large amount of lactic acid (Chen et al., 2017). Compared to other organic acids, lactic acid is a rather strong organic acid. Consequently, an abrupt decrease in pH value has been observed, resulting in better preservation of nutrients and better quality of silage fermentation (Muck et al., 2018). Therefore, LAB are often used in controlled fermentations to obtain a high quality silage product and to inhibit the growth of undesirable microorganisms (Carvalho et al., 2021). Among the different strains of LAB used in our study, inoculation of *L. plantarum* J39 showed significantly higher lactic acid content, resulting in significantly lower pH in Napier grass silage. Our observation was consistent with previous reports on forage fermentation that *L. plantarum* can rapidly convert fermentable sugars to lactic acid and exhibits higher acid tolerance during the ensiling (Alhaag et al., 2019; Bai et al., 2021).

Although starter cultures had no effect on lowering pH of whole-plant corn silage, they were important in Napier grass silage fermentation. The differences in plant material, including moisture, water-soluble carbohydrate (WSC), and epiphytic bacterial flora (Khota et al., 2016; Pholsen et al., 2016; Jiang et al., 2020; Yang et al., 2020) could lead to different fermentation characteristics of the two crops. In this study, the whole-plant corn contained higher starch and sugar content compared to Napier grass. The higher content of WSC in whole-plant corn, which provided sources of nutrient utilization, could promote microbial growth, including epiphytic lactic acid bacteria (LAB) (Yang et al., 2020). Whole-corn plant silage achieved high-quality scores in all treatments, including the non-inoculated control group. In contrast, higher silage quality scores were observed in the inoculated Napier grass groups than in the non-inoculated Napier grass silage. Based on microbiome analysis, we found that the whole-corn plant had a higher LAB abundance (phylum Firmicutes) than Napier grass. In Napier grass silage, Cyanobacteria and Proteobacteria were the predominant phyla in the initial phase of all treatments (D0). This result is similar to the previous study in which Cyanobacteria were found to be the dominant microorganisms before ensiling of three different plant species (Paspalum, White Popinac, and Stylo) (Li et al., 2019). Due to the different epiphytic bacterial communities, the whole-plant corn was more easily fermented into high-quality silage than Napier grass without starter inoculation. Our results suggest that the LAB starter inoculum may be required differently in the ensiling of different plant materials. Previous studies reported similar results where starter cultures inoculation could be able to improve the fermentation quality of grasses and legumes but not in corns, sorghum and sugar canes (Oliveira et al., 2017).

The addition of starter cultures could inhibit undesirable microorganisms and improve the fermentation quality of silage (Kim et al., 2021). The genus *Enterobacter*, which is known to cause silage spoilage (Oude Elferink et al., 2000; Li and Nishino, 2013), was found in greater abundance in the non-inoculated Napier grass silage than in the LAB inoculated samples. The use of different starter cultures contributed to a different decrease in the relative abundance of *Enterobacter* in the fermentation of Napier grass silage. The use of *L. plantarum* and/or *P. pentosaceus* as starter cultures clearly proved to be more efficient in reducing the relative abundance of *Enterobacter* compared to the use of *L. brevis* alone or without starter culture. This was confirmed by the faster pH drop and accumulation of lactic acid production in the T1, T3, and T4 treatment groups. Consequently, the growth of *Enterobacter* could be inhibited at lower pH (Heron et al., 1993), and an abundance of LAB could compete with *Enterobacter* for nutrients during fermentation (Pahlow et al., 2003; Muck, 2010), resulting in a lower population of *Enterobacter* population in the inoculated treatments compared to the non-inoculated treatment. In addition, a higher relative abundance of *Enterobacter* was observed in the fermentation of Napier grass silage with *L. brevis* inoculation (T2) and the control group than in the other groups. This was also supported by a slower pH drop and higher acetic acid content in both treatments. It has been reported that the growth of *Enterobacter* correlates with high levels of acetic acid production in king grass, paspalum grass and stylo legume silage (Li et al., 2019). In addition, Nishino et al. (2012) showed a correlation between *Enterobacteria* and high acetic acid concentration in guinea grass silage.

The epiphytic bacterial profile of each plant silage source differed in both diversity and relative abundance, which could be influenced by plant host-microbial interactions and environmental factors such as climate and farming practices (Dastogeer et al., 2020). Here, a higher baseline level of epiphytic LAB population in whole-plant corn showed higher abundance than that of Napier grass. In the absence of LAB inoculants, better fermentative quality was found in whole-plant corn silage than Napier grass silage. The Napier silage quality could be improved by addition of LAB inoculum. In addition to the type of epiphytic bacteria, the amount of WSC can also affect fermentation quality of silage. However, when comparing the quality of ensiled plants with different WSC, the WSC content has no significant influence on the chemical composition of the silage as long as lactic acid fermentation has taken place (Amer et al., 2012). Therefore, the use of LAB starter cultures was more important for successful silage production of plant material with lower natural LAB flora such as Napier grass than for the whole-plant corn. This study, which combined chemical analysis to determine silage fermentation quality with microbiota analysis to identify bacterial profiles during the fermentation process, reconfirmed the importance of identifying, properly selecting and using appropriate start cultures with the raw materials of choice for successful and cost-effective silage production.

## Conclusion

Preservation of green forage into silage is important for livestock feeding. However, under certain conditions, populations of epiphytic LAB are not always large enough or do not have the appropriate composition to promote efficient silage fermentation. LAB inoculants have become the most important additive for improving silage quality. The effects of different LAB strains as starter cultures and epiphytic bacterial flora needed to be explored to optimize the preservation of different raw materials. In this work, we compared fermentation quality and bacterial dynamics in two plant materials, whole-plant corn and Napier grass, with and without starter inoculation. The efficacy of *L. plantarum* J39, *L. brevis* BCC42336, and *P. pentosaceus* TBRC7603 as starter cultures were also compared in the silage system. The chemical analysis to determine the fermentation quality and metagenomic analysis to identify the bacterial community during ensiling were analyzed. Our study showed that the epiphytic LAB, associated with Napier grass, were much lower than those of whole-plant corn before ensiling. Chemical analysis showed that the use of LAB starter cultures was more important for the successful ensiling of Napier grass than for whole-plant corn. In addition, the fermentation quality of Napier grass could be improved by adding LAB inoculants, especially *L. plantarum*. Our results confirm the importance of identifying suitable starter cultures, understanding the natural flora of epiphytic LAB in plant, and applying them together to optimize cost-effective silage production.

## Data Availability Statement

The datasets presented in this study can be found in online repositories. The names of the repository/repositories and accession number(s) can be found below: https://www.ncbi.nlm.nih.gov/, PRJNA714904.

## Author Contributions

SS conceived, designed, and supervised the study. NJ collected sample. PA and NJ prepared DNA library for sequencing. NJ and SS determined fermentation quality. TU and WR analyzed microbiome data. SS, VP, and WR interpreted the data. NJ, TU, and WR wrote the manuscript. VP, SY, SS, and WR approved the final version of the manuscript. All authors read and approved the submitted version of the manuscript.

## Conflict of Interest

The authors declare that the research was conducted in the absence of any commercial or financial relationships that could be construed as a potential conflict of interest.

## Publisher’s Note

All claims expressed in this article are solely those of the authors and do not necessarily represent those of their affiliated organizations, or those of the publisher, the editors and the reviewers. Any product that may be evaluated in this article, or claim that may be made by its manufacturer, is not guaranteed or endorsed by the publisher.
