# Supplementary material for: Comparison of the Effects of Microbial Inoculants on Fermentation Quality and Microbiota in Napier Grass (Pennisetum purpureum) and Corn (Zea mays L.) Silage
Source: Front Microbiol. 2022 Jan 20;12:784535. doi: 10.3389/fmicb.2021.784535 (PMC8811201; doi:10.3389/fmicb.2021.784535)

**Figure S1.** The overview of experimental design. Whole-plant corns or Napier grasses were freshly collected for ensiling. The treatment groups were a non-inoculated control group (Control), and the groups inoculated with following starter inocula, *Lactobacillus plantarum* J39 (T1), *L. brevis* BCC42336 (T2), *Pediococcus pentosaceus* TBRC7603(T3), and a combination of *L. plantarum* J39, *L. brevis* BCC42336 and *P. pentosaceus* TBRC7603 (T4). Silage was sampling in triplicates and pooled together on day 0, day 3 and day 7 of ensiling for chemical and microbiota analyses.


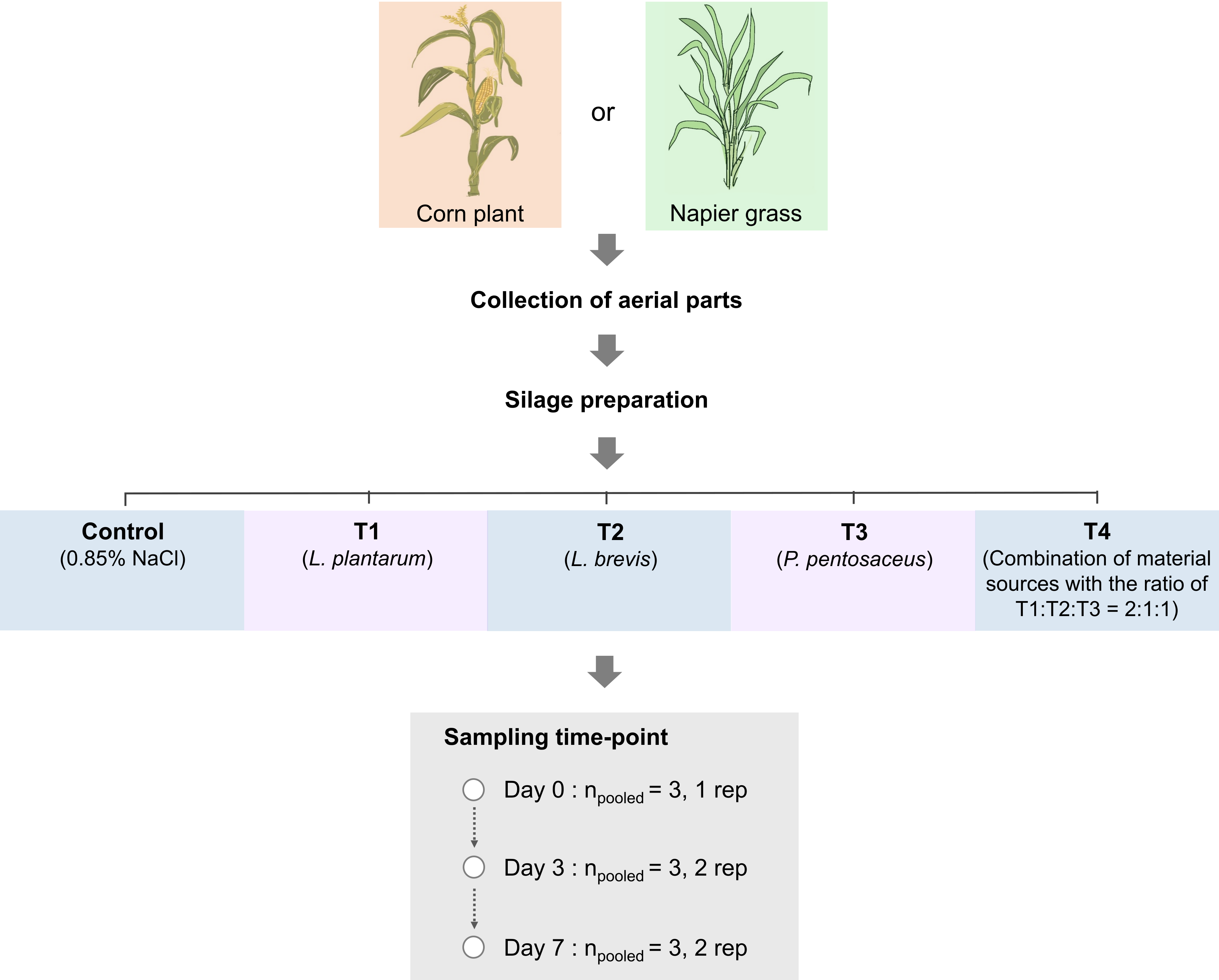

Supplement: Supplementary file 1 [file Data_Sheet_1.DOCX]
