# Supplementary figures and images for: Comparison of the Effects of Microbial Inoculants on Fermentation Quality and Microbiota in Napier Grass (Pennisetum purpureum) and Corn (Zea mays L.) Silage
Source: Front Microbiol. 2022 Jan 20;12:784535. doi: 10.3389/fmicb.2021.784535 (PMC8811201; doi:10.3389/fmicb.2021.784535)

**Figure S2.** Rarefaction curve analysis of all DNA libraries.

**
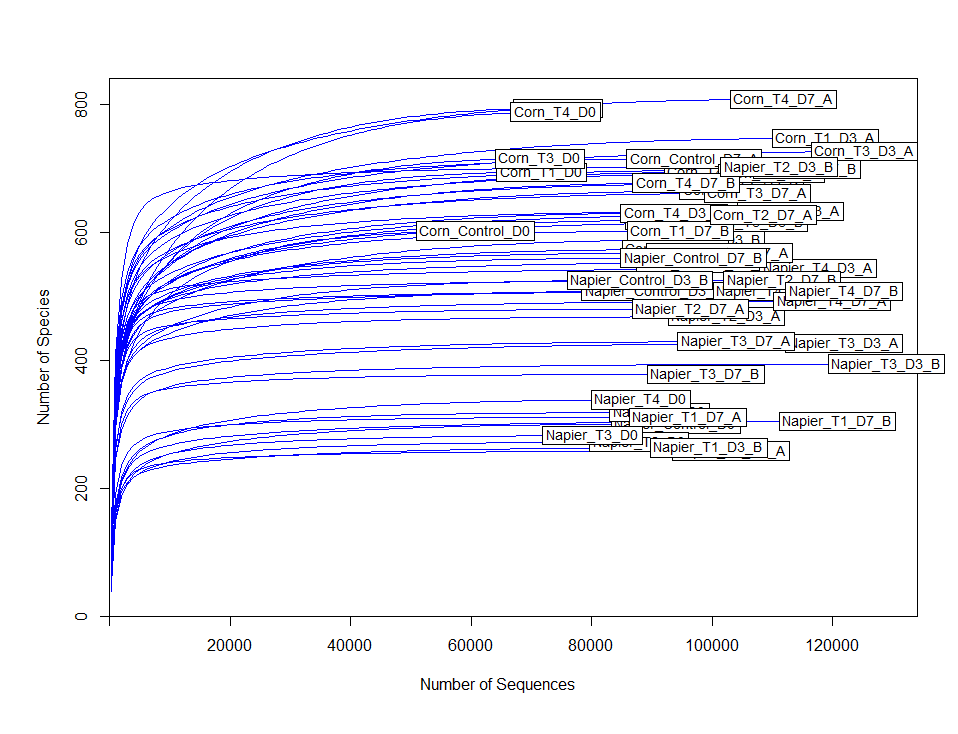
**

Supplement: Supplementary file 2 [file Data_Sheet_2.DOCX]
